# Seasonal dynamics of *Culicoides* (Diptera: Ceratopogonidae) biting midges, potential vectors of African horse sickness and bluetongue viruses in the Niayes area of Senegal

**DOI:** 10.1186/1756-3305-7-147

**Published:** 2014-03-31

**Authors:** Maryam Diarra, Moussa Fall, Assane G Fall, Aliou Diop, Momar Talla Seck, Claire Garros, Thomas Balenghien, Xavier Allène, Ignace Rakotoarivony, Renaud Lancelot, Iba Mall, Mame Thierno Bakhoum, Ange Michel Dosum, Massouka Ndao, Jérémy Bouyer, Hélène Guis

**Affiliations:** 1 Institut Sénégalais de Recherches Agricoles, Laboratoire National de l’Elevage et de Recherches Vétérinaires, Dakar, Sénégal; 2 Université Gaston Berger, Laboratoire d’Etudes et de Recherches en Statistiques et Développement, Saint-Louis, Sénégal; 3 Cirad, UMR15 CMAEE, F-34398 Montpellier, France; 4 INRA, UMR1309 CMAEE, F-34398 Montpellier, France; 5 Faculté des Sciences et Techniques, Département de Biologie Animale, Université Cheikh Anta Diop, Dakar, Sénégal

**Keywords:** Vector-borne disease, Insect vectors, Spatial and temporal dynamics, Light traps, *Culicoides oxystoma*, *Culicoides imicola*, Africa, Arbovirus, Orbivirus, Equids

## Abstract

**Background:**

The African horse sickness epizootic in Senegal in 2007 caused considerable mortality in the equine population and hence major economic losses. The vectors involved in the transmission of this arbovirus have never been studied specifically in Senegal. This first study of the spatial and temporal dynamics of the *Culicoides* (Diptera: Ceratopogonidae) species, potential vectors of African horse sickness in Senegal, was conducted at five sites (Mbao, Parc Hann, Niague, Pout and Thies) in the Niayes area, which was affected by the outbreak.

**Methods:**

Two Onderstepoort light traps were used at each site for three nights of consecutive collection per month over one year to measure the apparent abundance of the *Culicoides* midges.

**Results:**

In total, 224,665 specimens belonging to at least 24 different species (distributed among 11 groups of species) of the *Culicoides* genus were captured in 354 individual collections. *Culicoides oxystoma*, *Culicoides kingi*, *Culicoides imicola*, *Culicoides enderleini* and *Culicoides nivosus* were the most abundant and most frequent species at the collection sites. Peaks of abundance coincide with the rainy season in September and October.

**Conclusions:**

In addition to *C. imicola,* considered a major vector for the African horse sickness virus, *C. oxystoma* may also be involved in the transmission of this virus in Senegal given its abundance in the vicinity of horses and its suspected competence for other arboviruses including bluetongue virus. This study depicted a site-dependent spatial variability in the dynamics of the populations of the five major species in relation to the eco-climatic conditions at each site.

## Background

*Culicoides* (Diptera: Ceratopogonidae) are small midges (1 to 3 mm) presenting a huge diversity with more than 1,300 species described worldwide [1,2] of which some 96% are hematophagous. The females of some species of *Culicoides* are the vectors of pathogens of humans and animals. However, the geographical distribution and pathogenicity of the disease agents transmitted to humans are limited, so the epidemiological relevance of *Culicoides* is primarily for animal health [3,4]. In addition to the African horse sickness virus (AHSV), *Culicoides* may also transmit bluetongue virus (BTV), epizootic haemorrhagic disease virus (EHDV), equine encephalitis virus (EEV), Akabane virus, bovine ephemeral fever virus and viruses in the Palyam group [5]. However, the two diseases with the greatest veterinary impact among these are undoubtedly bluetongue (BT) and African horse sickness (AHS). BT and AHS are caused by orbiviruses mainly affecting ruminants and equids respectively. The significance of these two arboviruses derives from their very broad geographical distribution, their potential for spreading rapidly and their major economic impact, all of which justify them being listed as notifiable diseases by the World Animal Health Organisation [6]. Since 1998, Europe has suffered a series of epizootic outbreaks of BTV with disastrous consequences on animal health [7-9].

BTV has been known in South Africa since at least the beginning of the 20th century [10]. Towards 1925, BTV was introduced into Senegal through infected sheep that were imported from South Africa. The disease continued its progression in the sheep sent to Richard-Toll (in Senegal) as the area was infested with the vectors [10]. Research on the epidemiological status of this disease in Senegal is limited to two studies conducted in the 1980s stating that seroprevalence varies between 30 and 59% among sheep and goats [11,12].

The mortality rate due to AHS can reach 90% in susceptible horses [13]. This disease has been recognised for several centuries and probably originates from Africa [14]. It is thought to have been described for the first time in Senegal at the end of the 19th century [15]. The latest epizootic of AHS in Senegal occurred in 2007. It caused considerable economic losses, estimated to 0.9 billion FCFA (1.4 million euros) [16]. On a national scale, this epizootic led to an estimated mortality rate of 0.2% and morbidity rate of 0.3% in traditional horse breeding farms, and a 5.4% mortality rate in modern stud farms [16,17].

More than 30 species of *Culicoides* have been recorded in Senegal [18-20]. However, most entomological studies go back a long time, are limited in scope and did not specifically target the species in the vicinity of the animals whose health could potentially be impacted. Thus, it appeared necessary to update the list of the *Culicoides* species present in Senegal prior to assessing their epidemiological relevance. The present study aimed to describe the seasonal dynamics of the main species present in the vicinity of horses, which may be potentially involved in the transmission of AHSV in the Niayes region of Senegal.

## Methods

### Survey sites

The survey was conducted at five horse stables in the Niayes region in the vicinity of Dakar and Thies. The Niayes region of Senegal is a 25- to 30-km wide coastal band stretching over 180 km from Dakar to the southern tip of the Senegal River Delta. The climate is oceanic, typically warm and humid with strong, relatively constant winds. Vegetation is diversified and mainly composed of steppe and shrub savanna. Vegetation cover is generally less than 50%, except in the tree plantations (mangoes, citrus), abundant in this area. The sites surveyed and numbers of livestock recorded are shown in Table 1. Distance between sites ranged from 10 to 53 kilometres.

**Table 1:** Geographic coordinates of study sites and the numbers of animals identified

**Site**	**Latitude**	**Longitude**	Animals within 1 km of the trap*
Mbao	14.7467	−17.3327	32 horses, 2 poultry, 3 goats
Parc Hann**	14.7283	−17.4298	50 horses, 30 ponies, 11 lions, 1 tiger , 3 hyena, 5 jackal , 6 antelopes, 21 crocodiles, 9 turtles, 7 pitons
Niague	14.8234	−17.2499	4 cattle, 3 sheep, 30 horses, 1 donkey, 109 poultry, 1 dog
Pout	14.7665	−17.0357	12 horses, 8 donkeys, 1,700 sheep, 1,580 cattle, 240 goats, 25 buffalos, 11,000 poultry and 150 ostriches
Thies	14.794	−16.95	2 sheep, 24 horses (including 19 temporarily there to be mated), 50 poultry, 13 rabbits, 1 dog

*All traps were placed in the immediate vicinity of the horses.

**Parc Hann includes a riding center and a zoo.

Two main seasons are found in Senegal: the rainy season (July to October) and the dry season, sub-divided into the cold dry season (November to February) and the hot dry season (March to June). In the Niayes region, the average monthly temperature in July/August fluctuates around 27.5°C in Dakar. From November to February, the maximum temperature is below 28°C while the minimum temperature is 18°C along almost the whole coast. Moderate Harmattan wind can increase temperatures up to 31°C in May and June. Rainfall in the Niayes rarely exceeds 350 mm/year. Occult condensation (such as the condensation of mist and fog on foliage), referred to as “heug” or mango rain, not measured by standard rain gauges, often occurs during the dry season (particularly from December to February). The closeness of the ocean drives the high relative hygrometry that prevails in this region, varying from 15% to 90% depending on the season and distance from the sea. This region is characterized by shallow groundwater creating temporary ponds and the presence of particular vegetation usually found in much more humid regions classified as belonging to the Guinean eco-climatic area.

This area presents an important potential for agricultural development in general and animal production (large and small ruminants, equids, pigs and poultry) in particular. Dairy production, for instance, is particularly developed in this area. As the area is densely populated, wildlife is very scarce; in particular there are no wild antelopes or wild equids.

### Trapping methods

*Culicoides* midge apparent abundance was monitored by collecting insects at five sites for three consecutive nights monthly over a year. At four sites, Mbao, Parc Hann, Pout and Thies, collections were done from July 2011 to June 2012. At the fifth site, Niague, collections were done from November 2011 to October 2012. At each of the five sites, two Onderstepoort light traps were placed at least 10 m apart. Traps were operated from before dusk to after dawn. Traps were positioned close to the horses (hung on a post or a tree in the vicinity of the animals) at a height of 1.5 to 2 m from the ground.

The NOAA (National Oceanic and Atmospheric Administration) data for daily rainfall were extracted for each of the 5 sites, (http://iridl.ldeo.columbia.edu/expert/SOURCES/.NOAA/.NCEP/.CPC/.FEWS/.Africa/.DAILY/.ARC2/.daily/.est_prcp/). Total monthly rainfall was calculated for each site. The MODIS (Moderate-resolution Imaging Spectroradiometer) 8-day temperature data, LSTday (Land Surface Temperature day) and LSTnight (Land Surface Temperature night), were retrieved for each site for the whole period of capture. At each site, we calculated the LST_mean between LSTday and LSTnight for the 8 days covering the days of capture.

### Identification of *Culicoides*

All the samples were first sorted to discard other insects than *Culicoides*. When the volume of the catch exceeded 6 ml, sub-samples of 3 ml of sedimented *Culicoides* were taken out of the overall sample in accordance with a modified Van Ark & Meiswinkel [21] procedure. All the specimens were morphologically identified under a dissecting microscope. As no key encompassing the whole West African *Culicoides* species diversity exists, several dichotomous keys or species descriptions [20,22-26] were used to identify the specimens collected. Gender was also recorded.

### Statistical analyses

The maximum abundance of both traps for three nights of collecting per month and per site was used for performing analyses, statistical tests and for plotting the graphs. Maximum numbers collected were preferred to mean numbers collected because the number of specimens collected in a trap can drop very quickly when local weather conditions are sub-optimal. Hence, the maximum of several consecutive light trap collections was considered as the best measure for abundance over a short time period [27].

A log10(n + 1) transformation was applied to abundance data of night catches. We then compared the transformed maxima between sites by performing a Kruskal-Wallis test [28]. All the statistical analyses were conducted using the R version 2.15.2 [29]. Site mapping was performed with the Quantum GIS software (2.0.1).

## Results

In the results below, we focus on the proven and potential vectors of BTV and/or AHSV because those were the most abundant at the trapping sites.

In total, 224,665 specimens of the *Culicoides* genus (181,464 females and 43,201 males) belonging to at least 24 different species (Table 2) were caught during the 354 separate light trap collections at 4 sites (Mbao, Pout, Thies and Parc Hann) from July 2011 to June 2012 and at Niague from November 2011 to October 2012. The most abundant species were *C. oxystoma*, *C. kingi*, *C. imicola*, *C. enderleini* and *C. nivosus* which accounted for respectively 43.3% (97,351), 34.6% (77,826), 12.5% (28,123), 4.2% (9,379) and 1.4% (3,047) of the total number of specimens collected. These 5 species accounted for 90% of the total capture (Table 2).

**Table 2:** **Numbers of *Culicoides *** **trapped in the Niayes area of Senegal**

**Sites**	**Mbao**			**Niague**			**Parc Hann**			**Pout**			**Thies**			**Total (% of total catch) (n = 354)**
	**(n = 70)**			**(n = 70)**			**(n = 70)**			**(n = 72)**			**(n = 72)**			
***Culicoides *** **species**	**F**	**M**	**freq**	**F**	**M**	**freq**	**F**	**M**	**freq**	**F**	**M**	**freq**	**F**	**M**	**freq**	
C. oxystoma*	60579	13603	94.3	1530	707	85.7	10589	3280	100	3087	887	100	2284	805	94.4	97351 (43.33)
C. kingi*	9062	3593	94.3	51803	12672	100	239	41	60.0	209	6	55.5	186	15	55.5	77826 (34.64)
C. imicola*	11328	2769	100	9446	643	100	459	93	94.3	1301	256	97.2	1642	186	100	28123 (12.52)
C. enderleini*	3617	204	88.6	2079	465	100	1812	24	77.1	802	104	77.7	252	20	55.5	9379 (4.17)
C. nivosus*	490	385	88.6	399	103	97.1	808	182	97.1	407	96	80.5	97	80	75.0	3047 (1.36)
***C. austeni***	2	-	5.7	1062	2	68.6	664	2	68.8	-	-	-	11	-	11.1	1743
***C. similis***	191	256	60.0	399	138	85.7	145	165	85.7	143	15	61.1	76	39	77.7	1567
***C. moreli***	222	148	82.85	440	39	68.6	5	6	11.4	43	18	27.7	14	8	27.7	943
***C. gambiae***	23	47	54.3	650	179	94.3	1	1	5.7	2	1	8.3	7	2	13.8	913
***C. bolitinos***	66	28	65.7	204	15	80.0	7	-	11.4	26	6	25.0	307	15	69.4	674
***C. distinctipennis***	126	71	65.7	142	69	80.0	101	61	82.8	12	1	19.4	2	-	-	585
***C. milnei***	6	-	8.6	20	-	22.8	463	5	45.7	-	-	-	-	-	5.5	494
***C. murphyi***	34	25	48.6	160	68	91.4	64	54	71.4	1	-	2.7	3	2	13.8	411
***Culicoides sp.***	68	24	48.6	166	42	34.3	7	1	8.6	10	3	19.4	40	37	61.1	398
***C. leucostictus***	124	86	85.7	12	27	42.8	16	13	40.0	56	21	44.4	23	16	50.0	394
***C. loxodontis***	4	7	8.6	25	2	22.8	6	2	14.3	12	3	11.1	140	21	38.8	222
***C. pseudopallidipennis***	6	28	22.8	14	7	28.6	-	-	-	1	6	13.8	52	28	52.7	142
***C. miombo***	38	3	17.1	18	2	11.4	-	-	-	4	1	8.3	21	-	16.6	87
***C. accraensis***	1	-	2.8	1	-	2.8	2	-	2.8	-	-	-	41	34	30.5	79
***C. pycnostictus***	15	11	31.4	9	2	22.8	5	1	8.6	13	1	25.0	8	5	19.4	70
***C. hortensis***	-	-	-	33	-	11.4	33	-	20.0	-	-	-	-	-	-	66
***C. pretoriensis***	1	16	5.7	-	-	-	-	-	-	-	-	-	18	19	22.2	54
***C. azerbajdzhanicus***	-	-	-	25	4	42.8	-	2	5.7	3	-	8.3	7	1	16.6	42
***C. translucens***	-	-	-	2	-	5.7	-	-	-	-	-	-	16	12	13.8	30
***C. dekeyseri***	-	-	-	17	8	28.6	-	-	-	-	-	-	-	-	-	25
**Total**	86003	21304		68656	15194		15426	3933		6132	1425		5247	1345		224665

Numbers of *Culicoides* captured during the whole trapping period (all nights, n = number of trap-nights) are presented by species and location (F = female, M = male, freq = frequency, n = number of trap-nights). *5 dominant species.

**Figure 1:**
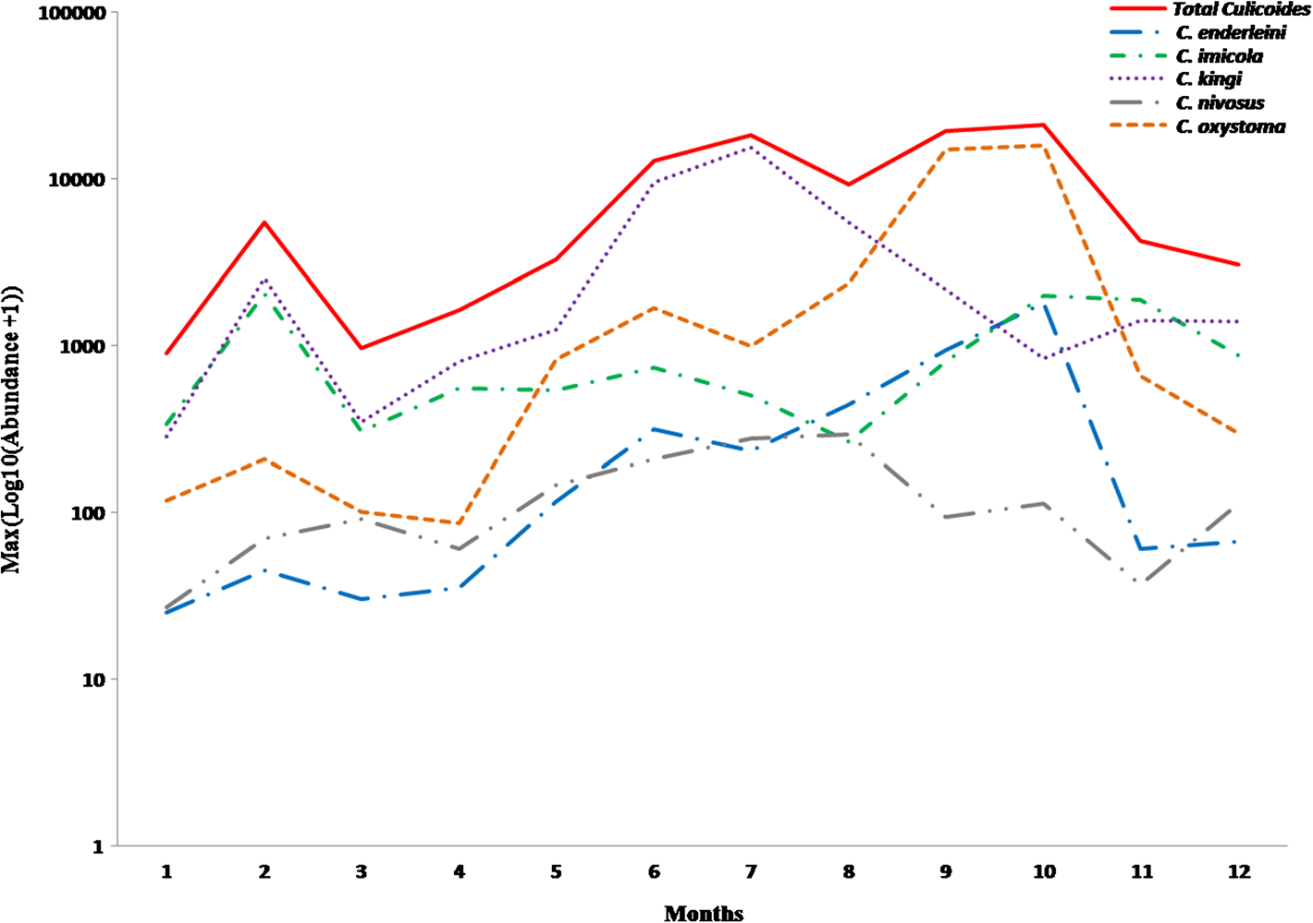
**Seasonal variations of *Culicoides *abundance in the Niayes area of Senegal.** Total abundance of *Culicoides* during the night of maximum catch is plotted on a log10 scale (in red line). For the 5 dominant species the sum of individuals caught at each site on the night of monthly maximum catch (by site and by species) are presented. Months’ quotation: 1 = January to 12 = December.

Total monthly abundance of *Culicoides* was computed as the sum of all species collected during the night of maximum catch for all sites. The peak was recorded in September and October (Figure 1). The apparent abundance peaks differ between the 5 abundant species. Peak of abundance for *C. oxystoma* was observed in September and October, in July for *C. kingi*, February for *C. imicola,* October for *C. enderleini* and August for *C. nivosus. Culicoides* apparent abundance differs significantly (p = 0.012) between months.

Overall, C*. kingi* was the most abundant species in terms of annual mean of maximum monthly abundance (695.0), followed by *C. oxystoma* (637.3), *C. imicola* (180.9), *C. enderleini* (68.6), *C. nivosus* (25.5); the mean of maximum monthly catches for the other species was less than 20 (Table 3). The species for which the mean of maximum monthly catches was highest at all sites was *C. oxystoma* with the exception of Niague, where it was superseded by *C. kingi*.

**Table 3:** Annual mean of maximum monthly catch in the Niayes area of Senegal

**Sites**	**Mbao**	**Niague**	**Parc Hann**	**Pout**	**Thies**	**Annual mean of maximum catch**
***Culicoides *** **species**	**(M ± SD)(n = 70)**	**(M ± SD)(n = 70)**	**(M ± SD)(n = 70)**	**(M ± SD)(n = 72)**	**(M ± SD)(n = 72)**	
C. kingi*	371.8 ± 402.5	3070.3 ± 4382.0	12.0 ± 16.9	13.3 ± 29.7	7.6 ± 13.5	695.0
C. oxystoma*	2221.5 ± 4384.1	106.3 ± 148.3	602.8 ± 1528.5	134.1 ± 187.3	121.9 ± 185.6	637.3
C. imicola*	332.6 ± 433.5	431.4 ± 436.9	26.8 ± 50.7	49.2 ± 35.3	64.8 ± 81.1	181.0
C. enderleini*	109.8 ± 159.5	106.6 ± 146.8	86.3 ± 241.5	27.1 ± 40.2	13.2 ± 31.4	68.6
C. nivosus*	28.0 ± 34.0	22.1 ± 21.2	45.9 ± 41.9	24.8 ± 25.2	6.8 ± 9.5	25.5
***C. austeni***	0.2 ± 0.4	46.9 ± 72.7	25.9 ± 40.8	-	0.7 ± 1.4	18.4
***C. similis***	17.2 ± 21.1	20.7 ± 20.9	12.3 ± 14.1	7.7 ± 10.2	4.3 ± 2.7	12.4
***C. gambiae***	3.0 ± 3.7	39.2 ± 53.0	0.2 ± 0.4	0.3 ± 0.4	0.5 ± 1.1	8.6
***C. milnei***	0.3 ± 0.6	1.4 ± 2.5	18.2 ± 37.5	-	-	6.6
***C. moreli***	10.7 ± 11.7	17.3 ± 30.0	0.3 ± 0.8	2.3 ± 4.3	0.9 ± 1.4	6.3
***C. distinctipennis***	7.5 ± 6.5	9.1 ± 6.5	6.5 ± 6.4	0.8 ± 1.0	0.2 ± 0.4	4.8
***C. murphyi***	2.7 ± 2.7	9.4 ± 7.6	5.3 ± 4.1	0.1 ± 0.3	0.3 ± 0.5	3.6
***C. bolitinos***	1.9 ± 1.8	5.5 ± 7.8	0.2 ± 0.6	1.2 ± 3.1	7.4 ± 13.8	3.2
***C. leucostictus***	6.3 ± 6.7	1.9 ± 1.8	1.3 ± 1.3	4.5 ± 7.2	1.8 ± 1.7	3.2
***C. hortensis***	-	2.7 ± 6.5	2.3 ± 4.9	-	-	2.5
***C. pseudopallidipennis***	2.5 ± 4.0	1.6 ± 2.1	-	0.5 ± 0.6	4.3 ± 4.6	2.2
***C. loxodontis***	0.9 ± 2.2	1.7 ± 2.1	0.4 ± 0.6	0.8 ± 1.8	7.1 ± 14.3	2.2
***C. dekeyseri***	-	1.7 ± 2.4	-	-	-	1.7
***C. pretoriensis***	0.8 ± 2.2	-	-	-	1.6 ± 3.6	1.2
***C. translucens***	-	0.2 ± 0.4	-	-	2.1 ± 4.7	1.1
***C. miombo***	1.7 ± 3.4	1.1 ± 2.8	-	0.3 ± 0.8	1.2 ± 2.5	1.1
***C. accraensis***	0.1 ± 0.3	0.1 ± 0.3	0.2 ± 0.6	-	3.2 ± 5.5	0.9
***C. pycnostictus***	1.6 ± 3.6	0.7 ± 0.8	0.4 ± 0.8	0.7 ± 0.9	0.9 ± 1.3	0.9
***C. azerbajdzhanicus***	-	1.4 ± 1.2	0.2 ± 0.4	0.2 ± 0.4	0.6 ± 0.8	0.6
**Mean of max catches**	**115.7**	**134.7**	**36.9**	**13.5**	**8.4**	**64.7**

The annual mean of monthly maximum abundance (M) ± standard deviation (SD) for each species during the whole trapping period are presented by location (n = number of trap-nights). The annual mean of maximum monthly abundance was the mean of the 12 maximum collections made at each site. *5 dominant species.

Throughout the study, the most abundant species at the trapping sites were also the most frequently collected. Taking all catches together, *C. imicola* was the most frequent species (98.4%), followed by *C. oxystoma* (95.6%), *C. nivosus* (89.0%), *C. enderleini* (82.4%), and *C. kingi* (76.9%). However, these occurrences varied according to the site: the occurrence of *C. imicola* was 100% at Mbao, Niague and Thies while the occurrence of *C. oxystoma* was 100% at Niague, Parc Hann and Pout (Table 2).

**Figure 2:**
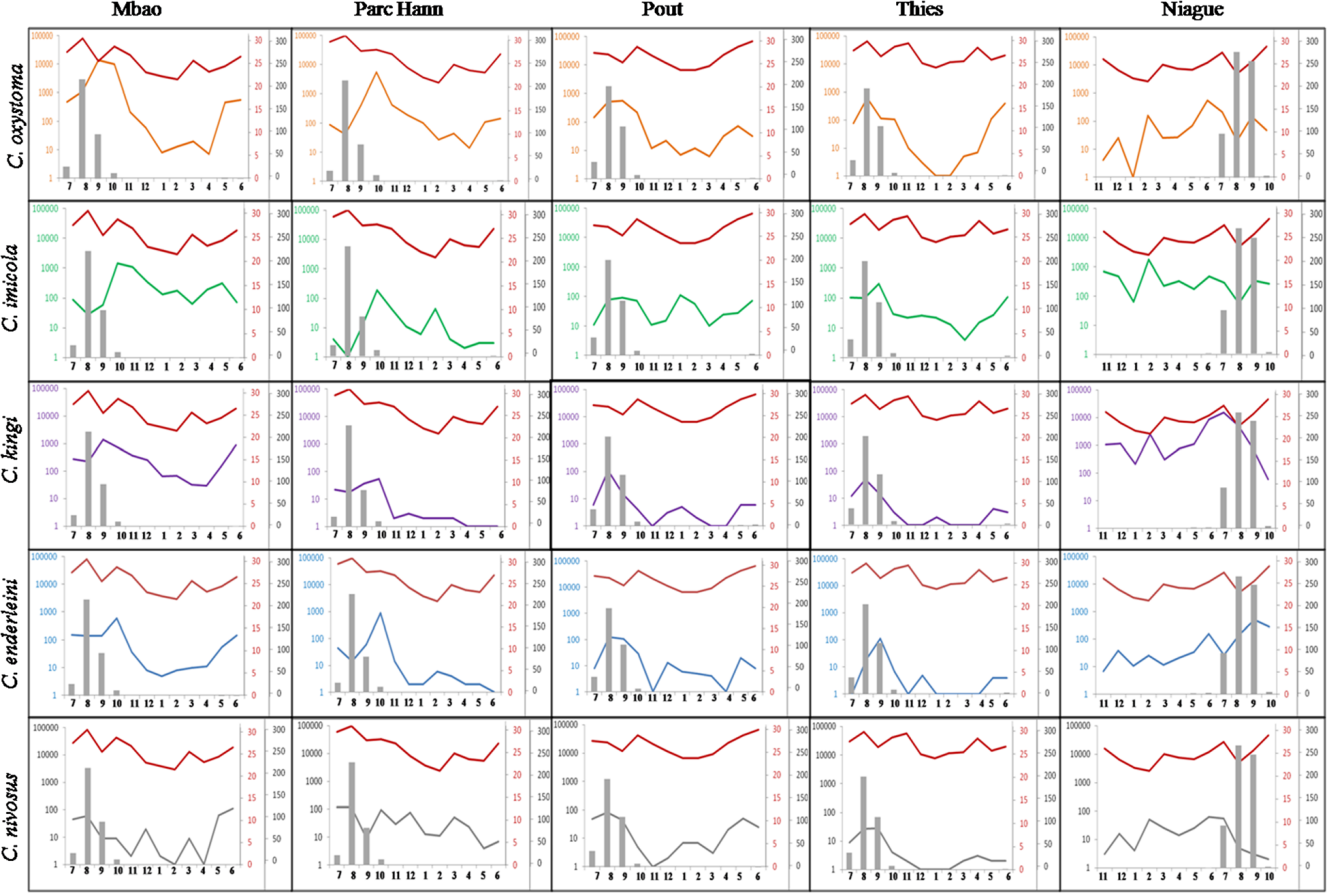
**Spatio-temporal variations of the monthly maximum catch of the 5 dominant species for each site.** Monthly rainfall quantities are indicated in grey. Note the log10 scale for the abundance of the monthly maximum catch. LST_mean during the period of catch for each site is plotted on the red line, expressed in degrees Celsius. The bold horizontal lines separate the species; the bold vertical lines separate the sites. Months’ quotation: 1 = January to 12 = December.

**Figure 3:**
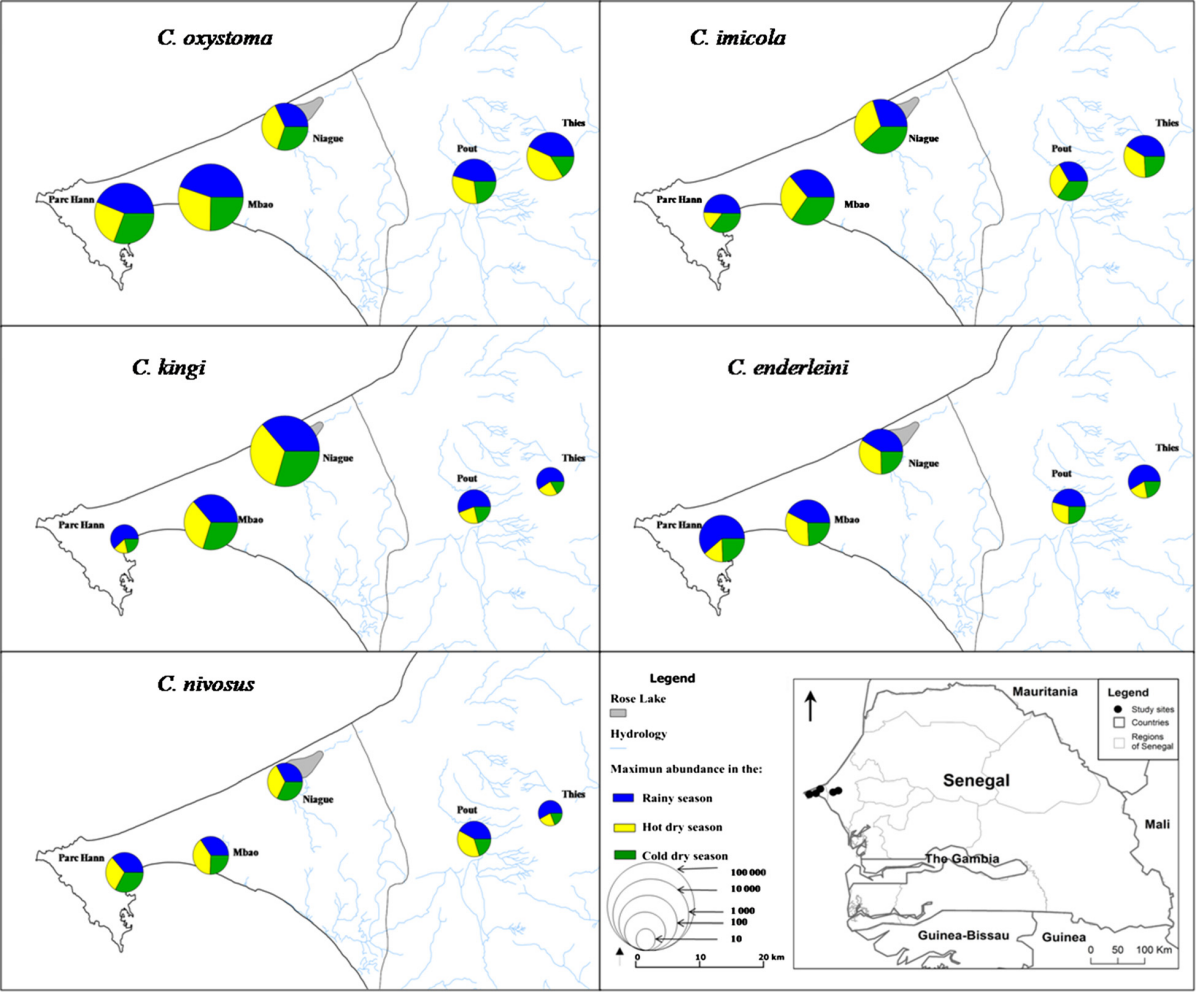
Spatial distribution of the 5 dominant species in the Niayes area of Senegal.

The rainy season began in July and ended in October with maximum rainfall in August 2011 and August 2012. The spatial and temporal variations for the 5 predominant species (Figure 2) showed that abundance peaks for *Culicoides* varied according to site and species. The maximum abundance was highly significantly different (p <10^−3^) between sites. The differences in maximum abundance between species were also very significant (p < 10^−3^). Spatial variations in maximum abundance within each season (rainy season, hot dry season and cold dry season) for the 5 predominant species are shown in Figure 3. The differences in maximum abundance between seasons were statistically significant (p < 10^−3^). At four of the five locations (Mbao, Parc Hann, Pout and Thies), the highest maximum abundance for *C. oxystoma*, *C. kingi* and *C. enderleini* were obtained during the rainy season when the LSTmean temperatures were the highest (25 to 30°C) (Figure 2). In contrast, during the cold dry season when LST_mean temperatures were the lowest (< 25°C), a decrease in *C. oxystoma*, *C. kingi* and *C. enderleini* abundance was observed at Mbao, Parc Hann, Pout and Thies (Figure 2). At Niague, a seasonal decline in the numbers of these species collected was not observed. At this location, largest numbers were collected during the hot dry season (March to June) for *C. oxystoma* (namely, in June - see Figure 2 for more details) and *C. nivosus*; during the cold dry season (November to February) for *C. imicola* (namely, February - see Figure 2)*.* At Pout, the abundance of *C. imicola* remained relatively stable, varying from 10 to 100 specimens per night all year round. It exhibited two small peaks of abundance: a peak during the rainy season and another in January. *C. nivosus* was highly abundant during the hot dry season (i.e., June - see Figure 3) at Mbao and Niague.

**Figure 4:**
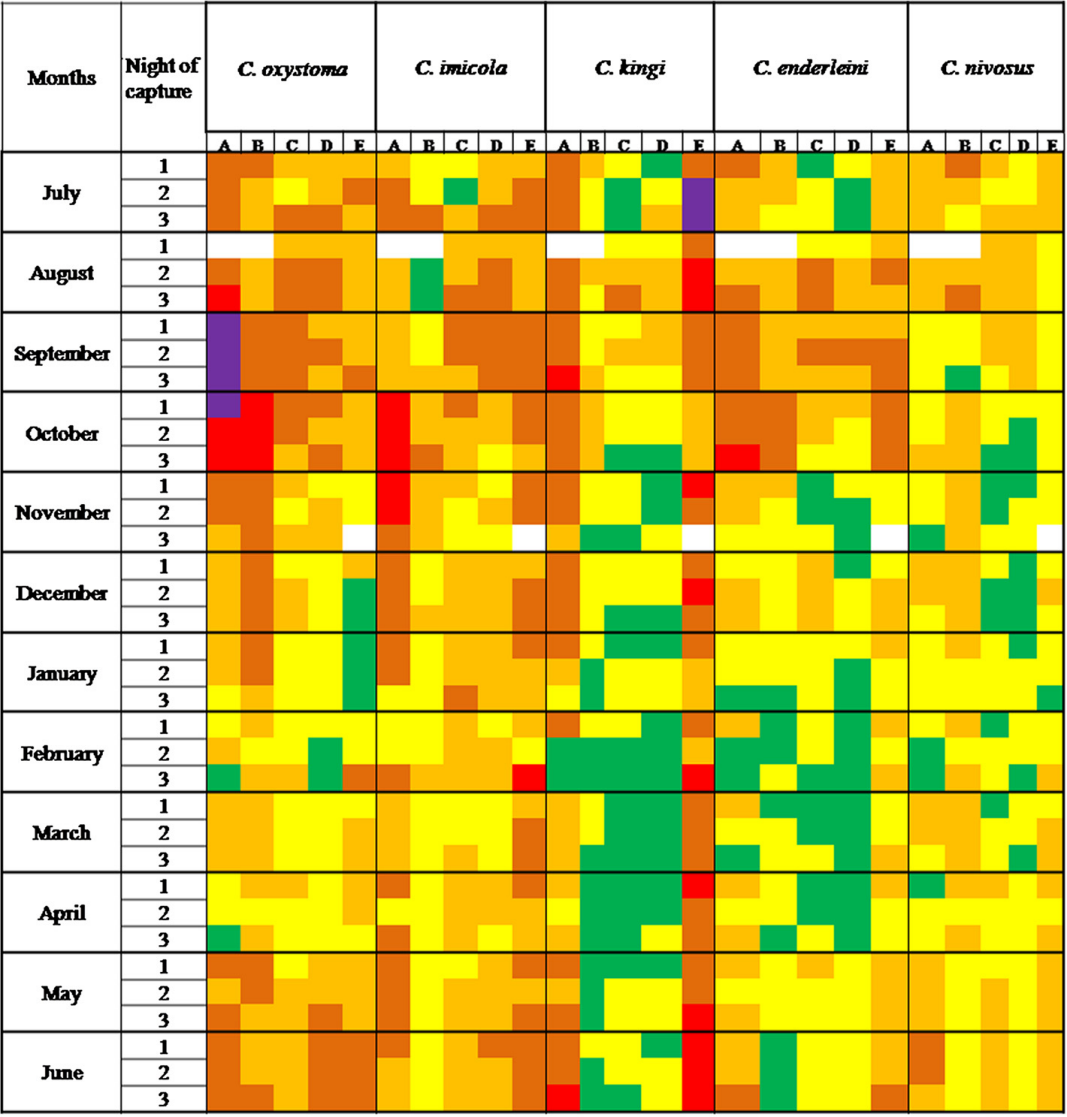
**Mondrian matrix of monthly *Culicoides *(3 nights per month) abundances.** Mondrian matrix of monthly *Culicoides* (3 nights per month) abundances (colour coded) for the 5 dominant species during 1 year (July 2011–June 2012 for Mbao **(A)**, Parc Hann **(B)**, Pout **(C)** and Thies **(D)**; November 2011-October 2012 for Niague **(E)** in Senegal. Green = 0 *Culicoides*, light yellow = 1–9, bright yellow = 10–99, orange = 100–999, red = 1,000-9,999, purple = 10,000-99,999, white = no data. The bold vertical lines separate the species; the bold horizontal lines separate the months.

The Mondrian matrix (term and figure proposed by Meiswinkel et *al.*[30]) shows the results of all the 3 nights of collecting for the 12 months in (Figure 4). It also shows a spatial and temporal heterogeneity of the 5 dominant species in the Niayes region. In this region, the activity of *C. oxystoma*, *C. imicola* and *C. nivosus* often persists all year round, with a few exceptions. *C. oxystoma* was absent for three consecutive nights of collecting in January at Niague, and likewise *C. nivosus* was absent for three consecutive nights of collecting in December at Thies. *C. kingi* and *C. enderleini* have longer periods of “apparent inactivity”. *Culicoides kingi* was absent in 8 of 9 consecutive nights of collecting from February to April at Pout and Thies, in 11 of 15 consecutive nights of collecting from February to June at Mbao. *Culicoides enderleini* was absent in 11 of 12 consecutive nights of collecting from January to April at Thies and 3 consecutive nights of collecting in June at Mbao (Figure 4).

## Discussion

Though the present study was conducted in a relatively small area of Senegal, we were able to confirm the presence of a number of *Culicoides* species recorded in previous surveys. Species not recorded in previous surveys in Senegal include: *Culicoides austeni, Culicoides azerbajdzhanicus, Culicoides bolitinos, Culicoides hortensis, Culicoides leucostictus, Culicoides loxodontis, Culicoides milnei, Culicoides miombo, Culicoides murphyi, Culicoides pretoriensis* and *Culicoides translucens*. Of 14 species of *Culicoides* collected in The Gambia by Rawlings *et al*. [31] with Pirbright incandescent light traps, 9 were also found in the Niayes region: *Culicoides distinctipennis*, *C. enderleini, C. imicola, C. leucostictus, C. milnei, C. miombo, C. nivosus, Culicoides pycnostictus* and *Culicoides similis*. The major species shared by The Gambia and Senegal was *C. enderleini* and *C. imicola.* For these two species, the lowest abundances were observed in December and in March/April both in The Gambia [31] and the Niayes region of Senegal (our work). The largest numbers of *C. kingi*, one of the 5 most abundant species in the Niayes, were collected in July. This species was also described in Sudan with abundance peaking in February and July [32].

In the past, several cases of AHS were reported in the Niayes region. In particular, the index case of the 2007 epizootic [17] was observed here. Indeed, horse numbers have increased significantly over the last years, with the settlement of private horse farms, in addition to already established horse-breeding farms. Furthermore, many seasonal horse cart drivers leave this area to join inner Senegal before the beginning of the rainy season, for agricultural works (ploughing, transportation). These seasonal migrations might have contributed to the spread of the AHS epizootic in 2007 [17]. Moreover, Parc Hann site includes a zoo and a riding centre situated within Dakar. Numbers of *Culicoides* collected at this site suggest that transmission could occur even in urbanized areas.

This study shows that 4 of the 5 dominant species recorded in the Niayes region are proven or suspected biological vectors of arboviruses: *C. oxystoma*, *C. kingi*, *C. enderleini* and *C. imicola*.

The first three of these species belong to the Schultzei group [18].*Culicoides oxystoma* is suspected of being a BTV vector in Asia and Australasia [33], and was found infected by bovine arboviruses in Japan (Akabane, Aino, Chuzan, and D’Aguilar) [34]. Non-engorged *C. kingi* and *C. enderleini* were found infected respectively by EHDV in Sudan [35], and BTV in South Africa [36]. Although viral isolation from field-caught specimens does not prove vector competence, it shows that the species will feed on infected hosts in field conditions. Indeed, in addition to feeding on infected hosts, two other conditions are required to define a species as a vector: allowing replication and transmission of the pathogen.

At least four species of the Imicola group were found at our five sites: *C. miombo*, *C. bolitinos*, *C. loxodontis*, and *Culicoides pseudopallidipennis. Culicoides imicola* is a proven vector of BTV [13,37,38] and AHSV [13,14,39], and suspected vector of EEV [40,41] and EHDV [42]. *Culicoides bolitinos* is a suspected vector of BTV [13,37,38], AHSV [14,39,43], EEV [40] and EHDV [42]. Lee [44] has shown that *C. miombo* is a suspected BTV vector. Furthermore, a very broad distribution of *C. miombo* has been reported in tropical and subtropical Africa, in particular in Zimbabwe, Botswana, Nigeria, Ivory Coast, The Gambia and South Africa [25].

The fifth most abundant species was *C. nivosus*. The abundance of this species was low in The Gambia [31] and in South Africa [45]. In the case of BTV, several studies have tested this species for its competence [46,47]; but until this date, *C. nivosus* has not been purported as a vector for BTV or AHSV. As far as we know, *C. pseudopallidipennis* was not incriminated of being either a BTV or AHSV vector.

Based on the major species collected, it appears essential to assess the vector competence for AHSV, the biting rate and host preferences of the species in the Schultzei group. Indeed, in the Niayes region, depending on their competence and capacity, *C. oxystoma*, and/or *C. kingi* and/or *C. enderleini* may be significant vectors, perhaps more so than those in the Imicola group. In any case, evaluating the competence and capacity of all the potential vector species is crucial to better assess the risk of transmission through space and time.

The results of the present study revealed one or two peaks of abundance for the species that are potentially involved in the transmission of AHSV (*C. oxystoma*, *C. imicola*, *C. kingi* and *C. enderleini)*. These peaks occurred from April to June, and from July to October, coinciding respectively with the emergence and spread of AHS in 2007 [17].

Previous observations showed that *C. imicola* was the dominant species in many sub-Saharan African countries: South Africa [48-50], Nigeria [51], Botswana [52], The Gambia [31] and Zimbabwe [53]. In Senegal, for the first time, this study shows the importance of *C. oxystoma,* which was the dominant species in terms of abundance at 4 of the 5 sites, and the second most abundant (behind *C. kingi*) at the fifth (Niague). *Culicoides imicola* was the second most abundant species at 4 sites and the 5th most abundant at Parc Hann. In terms of frequency of collection, *C. imicola* has a slight advantage over *C. oxystoma*.

The differences in abundance and frequency observed between sites for the 5 most abundant livestock associated species may be related to the ecological conditions that prevailed there, in particular the available larval habitats, i.e. humid locations with plenty of decaying organic matter such as animal droppings [54]. The high abundance of *C. oxystoma* at Mbao and Parc Hann may be connected with the presence of a permanent stream close to the Mbao riding center and a lake surrounded by marshy areas around Parc Hann [55]. The capacity of *C. kingi* larvae to develop in very salty muds exposed to sunlight [20] could contribute to explain why it is the most abundant species in Niague, a site close to a salt-saturated lake (Rose Lake, see Figure 2) The highest abundance for *C. nivosus* was observed at Mbao, perhaps in connection with the closeness of this site to the Mbao protected forest, as this species has been reported in other forested areas in Senegal (such as the Forest of Bandia) [19]. For many species of *Culicoides*, larval habitats remain poorly described and larval identification techniques limited. Furthermore, although larval habitats are important determinants of the presence and abundance of *Culicoides*, other factors such as host characteristics (species, numbers, distance) [56,57] or trapping methods [58-60] can also greatly influence the diversity and abundance of collections made.

September and October, i.e., the end of the rainy season, were the months of highest overall abundance of *Culicoides*. The highest abundances of *C. oxystoma* were recorded during the rainy season, namely August to October. The abundances of *C. kingi* declined considerably midway through the rainy season. This is possibly due to the fact that during the tropical winter season the salt content of the large body of briny water located close to the Niague riding center might decline. Likewise, a drop in abundance during the rainy season was also observed for *C. nivosus.*

*Culicoides* catches were made in the Niayes region of Senegal, i.e. a tropical environment, it is not surprising that activity of some dominant species is observed all year round, in contrast with what is found in temperate environments such as the Netherlands for instance [30]. The fact that vector species such as *C. imicola*, or potential vector species such as *C. oxystoma*, are active year round contributes to increasing the risk of transmission of BTV and AHSV in these areas since the length of the transmission season is increased and no overwintering mechanisms are necessary to ensure viral transmission the following year.

Overall, it is not easy to distinguish a stable pattern over time for each species as illustrated by the spatial and temporal variability displayed on the Mondrian matrix. There are important variations in dynamics between sites and between species: on a single site there are differences according to species, while for a single species, the pattern differs depending on the site, even for sites which are close to one another. As expected for a given site, we observed differences in patterns between species. In particular in Niague, the patterns observed for the 5 dominant species seemed to differ from those in the 4 other sites. Models need to be developed so as to better understand the influence of environmental and climatic parameters on the distribution and dynamics of *Culicoides* in Senegal.

## Conclusion

This descriptive study of the spatio-temporal dynamics of *Culicoides* (Diptera: Ceratopogonidae) was able to inventory the various species present in the Niayes region in Senegal and their abundance pattern over the course of the year. Very high abundances of the species are observed mainly in September and October in this region, particularly for *C. oxystoma*, *C. kingi*, *C. imicola*, *C. enderleini* and *C. nivosus,* which are the most abundant and frequent species at the trapping sites and exhibit differing spatial patterns. The investigation showed up the presence of several species including *C. oxystoma,* which may be potential vectors for BTV and AHSV, possibly calling into question the role of *C. imicola,* which is thought to play as the main vector in Africa. It is essential to assess their respective competence for these two viruses so as to better evaluate the risk of African horse sickness and bluetongue disease in Senegal.

## Abbreviations

AHSV: African horse sickness virus; BTV: Bluetongue virus; EEV: Equine encephalosis virus; EHDV: Epizootic haemorrhagic disease virus; LSTday: Land surface temperature day; LSTnight: Land surface temperature night; LST_mean: Land surface temperature mean.

## Competing interests

The authors declare that they have no competing interests.

## Authors’ contributions

HG, AGF, MTS and JB designed and supervised the study. MF, AGF, IM performed sampling and global management of the entomological material. RL extracted Modis data. MF and IM performed the species identification with the help of CG, TB, XA, and IR. MTB, AMD, MD and MN participated in field and laboratory activities. MD, AGF, MTS and HG analysed the data and wrote the first draft of the manuscript. All authors revised and approved the final version of the manuscript.
